# Improved Omnidirectional Odometry for a View-Based Mapping Approach

**DOI:** 10.3390/s17020325

**Published:** 2017-02-09

**Authors:** David Valiente, Arturo Gil, Óscar Reinoso, Miguel Juliá, Mathew Holloway

**Affiliations:** 1 System Engineering and Automation Department, Miguel Hernández University, Elche (Alicante) 03202, Spain; arturo.gil@umh.es (A.G.); o.reinoso@umh.es (Ó.R.); 2 Q-Bot Ltd., Riverside Business Park, London SW18 4UQ, UK; miguel@q-bot.co; 3 Dyson School of Design Engineering, Imperial College, London SW7 1NA, UK; mat@q-bot.co

**Keywords:** visual odometry, omnidirectional images, visual SLAM, feature matching, mapping

## Abstract

This work presents an improved visual odometry using omnidirectional images. The main purpose is to generate a reliable prior input which enhances the SLAM (Simultaneous Localization and Mapping) estimation tasks within the framework of navigation in mobile robotics, in detriment of the internal odometry data. Generally, standard SLAM approaches extensively use data such as the main prior input to localize the robot. They also tend to consider sensory data acquired with GPSs, lasers or digital cameras, as the more commonly acknowledged to re-estimate the solution. Nonetheless, the modeling of the main prior is crucial, and sometimes especially challenging when it comes to non-systematic terms, such as those associated with the internal odometer, which ultimately turn to be considerably injurious and compromise the convergence of the system. This omnidirectional odometry relies on an adaptive feature point matching through the propagation of the current uncertainty of the system. Ultimately, it is fused as the main prior input in an EKF (Extended Kalman Filter) view-based SLAM system, together with the adaption of the epipolar constraint to the omnidirectional geometry. Several improvements have been added to the initial visual odometry proposal so as to produce better performance. We present real data experiments to test the validity of the proposal and to demonstrate its benefits, in contrast to the internal odometry. Furthermore, SLAM results are included to assess its robustness and accuracy when using the proposed prior omnidirectional odometry.

## 1. Introduction

In the field of mobile robotics the problem of SLAM entails a demanding task which requires the simultaneous accomplishment of map building and robot estimation. This aspect poses a challenge when it comes to the complexity associated to the incremental nature of the process. In this context, the presence of non-linearities induces undesired injurious effects that may gravely aggravate and jeopardize the final estimation. In this sense, the internal odometer of the vehicle may be considered as a problematic source of non-linear noise [1]. Thus using the odometry data as a first prior input implies extra expenses for the system in order to obtain and maintain the convergence of the final estimation [2].

To date, great efforts have been made on the modeling of the odometry of the vehicle [3,4]. They have concentrated on different subfields of research. In [5], least squares calibration is used; estimation techniques such as back-propagation and neural networks in [6]; GPS data fusion with internal odometry data in [7,8].

Despite the large amount of research on odometer-related data techniques, nowadays, the visual sensors have emerged as a promising alternative with potential advantages such as low cost, light weight and low consumption. These sensors represent the basis for visual odometry, which has become a very convenient technique to recover the motion of a vehicle between two consecutive poses. In this sense, many applications have exploited the use of different kind of cameras: stereo-based [9,10,11,12], monocular-based [13,14,15] and omnidirectional-based [16,17,18,19]. The combination of different visual information [20] with several estimation algorithms [21,22,23] and fused sensor data [24,25,26], are some of the main aspects that sustain and reinforce the increasing growth of visual odometry. It proves to be a suitable method for the motion recovery of a vehicle within an endless list of possible applications in the framework of mobile robotics. Several studies on performance [27,28] have confirmed these potentials.

Other approaches have embedded visual odometry into final SLAM applications, such us [29], where the monocular-based version has been adapted to stereo [30] and recently to omnidirectional [31]. A novel tracking with depth values are fused into a SLAM estimation with 3D recovery. In this context, the work presented in this article proposes a 2D omnidirectional visual odometry approach; however, this can be conceived to be exploited solely in a real-time oriented system. Nonetheless, we have also assessed this visual odometry under an extended framework, that is, by evaluating improvements and performance of a view-based SLAM system, which exploits a dual 2D-3D capability to represent the environment. We intend to generate a reliable feed-forward input which can mitigate the instabilities induced by the internal odometer of a mobile robot. To that purpose, the current uncertainty of the system has been considered so as to be propagated to the matching process. Furthermore, we adapt the epipolar constraint as the key tool to extend the feature matching process to our sensor geometry, that is, to the omnidirectional reference system. This contributes to the robustness of a reinforced adaptive matching process which considerably reduces false positive. Thus the stability of the motion recovery computation between poses of the robot is enhanced. This aspect is also of paramount importance when dealing with the computation of motion recovery, which becomes especially sensitive when it is only sustained by omnidirectional measurements. Besides this, it represents a crucial stage as the basis of the observation model embedded within our SLAM approach.

In particular, we present an omnidirectional visual odometry that can be integrated into a general-purpose mobile robotic vehicle with catadioptric systems in order to aid in the indoor localization tasks within the framework of SLAM. The main contributions can be listed as follows:

- Adaption of the epipolar constraint to the reference system of an omnidirectional camera sensor.
- Propagation of the current uncertainty to produce an improved adaptive matching process.
- Reliable approach to motion recovery with several variants aiming at the improvement of performance.
- Fusion into a dual view-based SLAM system, as the main prior input in detriment of the internal odometry.

The remainder of this paper is structured as follows: Section 2 devises the main considerations to design the omnidirectional visual odometry. In particular, it presents the epipolar constraint adaption, the motion recovery procedure and the uncertainty propagation to obtain the adaptive matching. Section 3 introduces a brief outlook to the view-based SLAM approach, being the liable target application of this omnidirectional visual odometry; Section 4 presents the omnidirectional visual odometry and SLAM experimental results obtained with real data environments. These experiments were designed to test the validity and reliability of this approach, concentrating on the performance and the accuracy but also on the SLAM estimation; Section 5 establishes a discussion on these results; Section 6 finally exposes the conclusions extracted from the outputs of this work.

## 2. Visual Odometry

**Figure 1:**
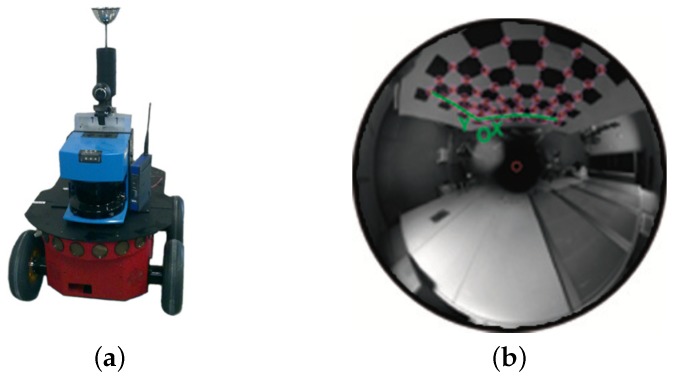
Real equipment used in this work: (**a**) Pioneer P3-AT mounted with an omnidirectional camera, internal odometer and laser range finder; (**b**) calibration procedure for the omnidirectional system.

**Figure 2:**
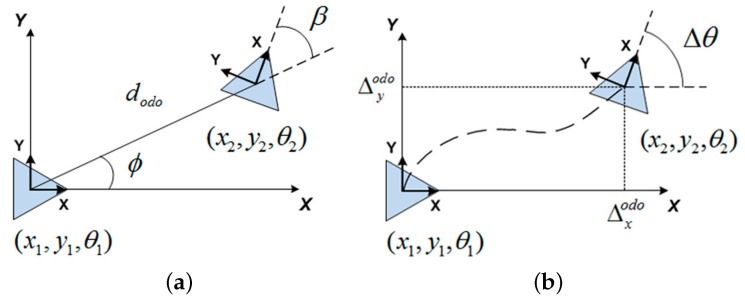
Visual odometry diagrams: (**a**) angular model; (**b**) differential model.

Visual odometry is generally agreed to be a relative camera motion recovering problem, which also implies motion recovery in the pose of the robot. The mobile vehicle used in this work, the Pioneer P3-AT, is presented in Figure 1a. It is a skid-steering four wheeled robot with two internal encoders [32] and kinematics model defined as [33]. Note that certain assumptions have to be made: (*i*) the mirror is a central system; (ii) the camera and mirror axes are well aligned; (iii) the mirror rotates symmetrically to the *z*-axis. For this reason, a specific calibration tool has been used [34], as shown in Figure 1b. Once said that, under these considerations, we only make use of the internal odometer, without the aid of any other mechanical sensor. Therefore, two of the most widely used models for such case are those incremental models presented in Figure 2. In particular, we concentrate on the angle-based relative motion, as observed in Figure 2a. Figure 2b shows the differential-based model.

Our omnidirectional visual odometry approach makes use of the matched points between consecutive omnidirectional views, captured at consecutive poses of the robot, at *t* and t+1: $\left(x_{1},y_{1},\theta_{1}\right)$ and $\left(x_{2},y_{2},\theta_{2}\right)$ respectively. We pursue the relative angles *β* and ϕ. Next, we take $d_{odo}$ given by the internal odometer as the initial scale guess, due to the monocular nature of the system. Note that we can also determine the scale factor by using visual recognition of patterns in the environment with well known dimensions. Therefore, we can proceed similarly to diagram shown in Figure 2a to infer the visual odometry model as:

(1) $$\left[\begin{array}{c} x_{2}\\ y_{2}\\ \theta_{2} \end{array}\right]=\left[\begin{array}{c} x_{1}\\ y_{1}\\ \theta_{1} \end{array}\right]+\left[\begin{array}{ccc} \cos(\phi )\; & \;0\; & \;0\;\\ \sin(\phi )\; & \;0\; & \;0\;\\ 0\; & \;1\; & \;1 \end{array}\right]\left[\begin{array}{c} d_{odo}\\ \beta \\ \phi \end{array}\right]$$

### 2.1. Epipolar Geometry

The introduction of epipolar geometry is essential when retrieving motion for visual odometry approaches based on feature matching. Here, we extend the planar epipolar constraint to our omnidirectional reference system. This allows to establish the fundamentals for the motion relation between omnidirectional images, and likewise to consecutive poses of the robot.

As stated in [35], the fundamental matrix matrix $F_{3\times 3}$ ∈ $R^{3}$ encapsulates the epipolarity as an intrinsic projective geometry between views, being only dependent on the camera calibration. Therefore, a given 3D point $X_{3\times 1}$ $\in R^{3}$, projects on different image reference systems, as *x* and $x^{\prime }$ in a first and second view respectively. Next, the image coordinates can be normalized through the essential matrix $E_{3\times 3}$ $\in R^{3}$ [36], with a known calibration matrix $K_{3\times 3}$ $\in R^{3}$:

(2) $$\begin{array}{c} x^{\prime T}Fx=0 \end{array}$$

(3) $$\begin{array}{c} E=K^{\prime T}FK \end{array}$$

(4) $$\begin{array}{c} {\hat{x}}^{\prime T}E\hat{x}=0 \end{array}$$

Next, the terms in *E* entail a general decomposition into a rotation $R_{3\times 3}$ and a translation $T=[t_{x},t_{y},t_{z}]$, by means of the skew symmetric ${\left[T\right]}_{x}$ [35]. Under the assumption of a 2D movement on the XY-plane, the relation is relaxed to:

(5) $$\begin{array}{cc} E={\left[T\right]}_{x}R & =\left[\begin{array}{ccc} 0 & 0 & \sin(\phi )\\ 0 & 0 & -\cos(\phi )\\ -\sin(\phi ) & \cos(\phi ) & 0 \end{array}\right]\left[\begin{array}{ccc} \cos(\beta ) & -\sin(\beta ) & 0\\ \sin(\beta ) & \cos(\beta ) & 0\\ 0 & 0 & 1 \end{array}\right]=\\ & =\left[\begin{array}{ccc} 0 & 0 & \sin(\phi )\\ 0 & 0 & -\cos(\phi )\\ \sin(\beta -\phi ) & \cos(\beta -\phi ) & 0 \end{array}\right] \end{array}$$

with the extra factor set by the lack of scale, being retrievable as mentioned above.

**Figure 3:**
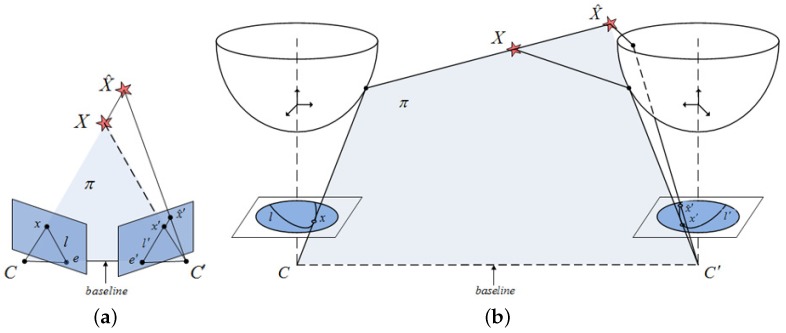
Epipolar constraint adaption: (**a**) planar reference system; (**b**) adaption to our omnidirectional reference system.

Figure 3 graphically compares the implications of the application of the epipolar constraint on the standard planar reference system, in Figure 3a, with our omnidirectional reference system, in Figure 3b. Now the 3D point *X* and its projection on two image planes, *x* and $x^{\prime }$, can be related through the coplanarity condition established by the epipolar plane, *π*, and the camera centers *C* and $C^{\prime }$. Notice the importance of *l* and $l^{\prime }$, as the epipolar lines resulting from the intersection of *π* with the image planes. They represent a potential advantage for the matching extraction, since $x^{\prime }$ is constrained to lie on $l^{\prime }$. Traditionally, this has been extensively used for matching purposes in stereo applications [37]. Similarly, we will define an adaptive matching process to predict matches by exploiting the epipolar line, now turned into an ellipse, as seen in Figure 3b for the omnidirectional reference system. It is also worth noting that this elliptical shape is the result of the intersection of *π* with the hyperboloid of two sheets that models our omnidirectional mirror.

### 2.2. Motion Recovery

Considering epipolarity on the omnidirectional reference system is crucial when dealing with the computation of the motion recovery between poses of the robot, especially when attempting to produce a robust prior input for navigating tasks, which is solely based on an omnidirectional camera.

**Figure 4:**
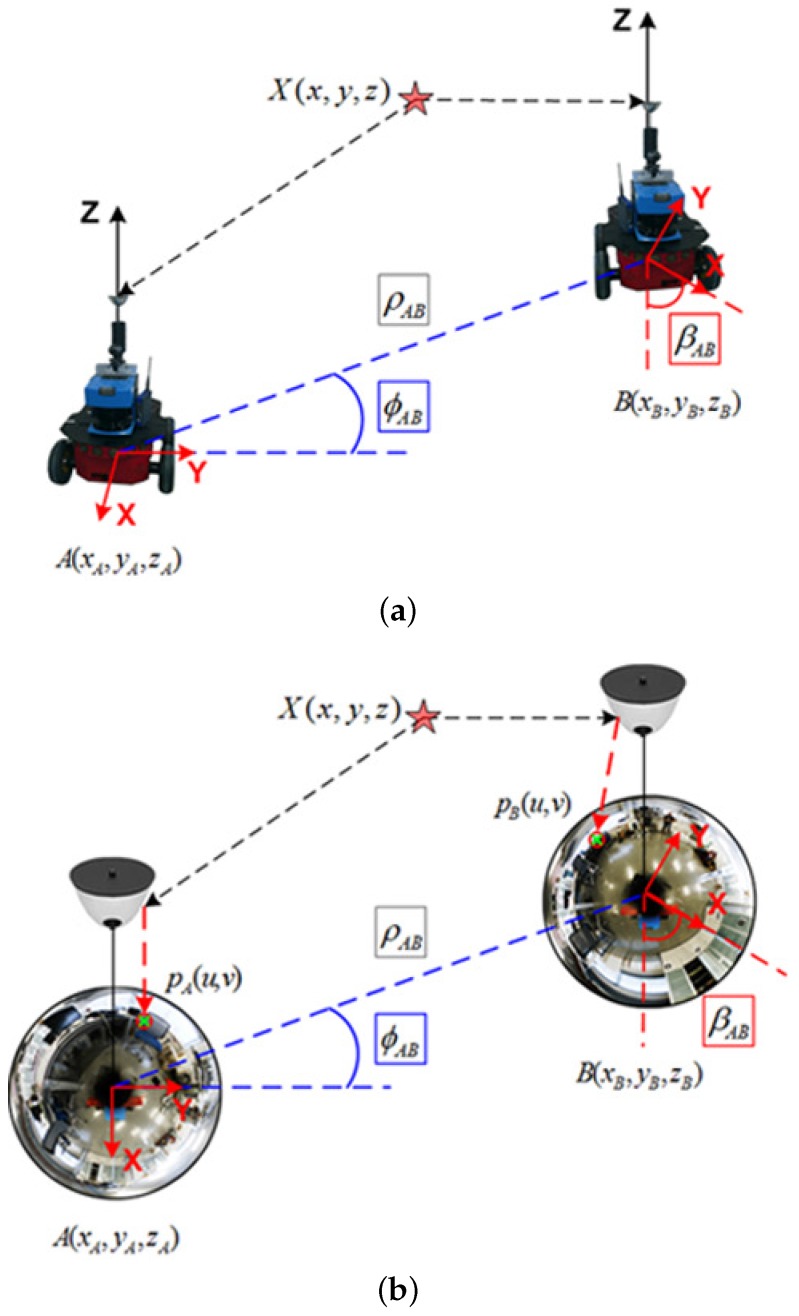
Motion recovery between poses *A* and *B*: (**a**) robot reference system; (**b**) analogous relation in the camera reference system. A 3D point, X(x,y,z) is indicated with its image projection (in pixels) on both camera frames, as $p_{A}\left(u,v\right)$ and $p_{B}\left(u,v\right)$, respectively.

Under this context, the motion relation can be defined as depicted in Figure 4, in terms of camera-to-pose equivalence. Notice that the inferred connections derived from the epipolar constraint in Figure 3 are transferred to Figure 4. Figure 4a,b present respectively, the same motion relation in both, the robot and the image reference systems. Then assuming that the camera rotates on the *z*-axis while it moves on the XY, and providing that Equation (4) is fulfilled, the problem can be explicitly relaxed to an only-XY movement, being now expressed in terms of unknown variables in the essential matrix, as $e=[e_{1},e_{2},e_{3},e_{4}]$:

(6) $$E=\left[\begin{array}{ccc} 0 & 0 & e_{1}\\ 0 & 0 & e_{2}\\ e_{3} & e_{4} & 0 \end{array}\right]$$

Therefore, Equation (4) can be linearly denoted as the system De=0, with *D* containing the coordinates coefficients $x=(x_{0},y_{0},z_{0})$ and $x^{\prime }=\left(x_{1},y_{1},z_{1}\right)$ for two matched points between views. Note that *D* is *N*x4, with *N* the total number of matched points found, being $N_{min}=4$.

(7) $$D_{i}=\left[\begin{array}{cccc} x_{0}z_{1} & y_{0}z_{1} & z_{0}x_{1} & z_{0}y_{1} \end{array}\right],\forall i\in \left[1,\ldots ,N\right]$$

Following [35], a SVD decomposition allows to retrieve the relative angles (*β*, ϕ) and thus the two possible translations and rotations as:

(8) $$\phi =atan\frac{-e_{1}}{e_{2}}=atan\frac{\sin(\phi )}{\cos(\phi )}$$

(9) $$\beta =atan\frac{e_{3}}{e_{4}}+atan\frac{-e_{1}}{e_{2}}=\left(\beta -\phi \right)+\phi$$

(10) $$\begin{array}{c} t_{x1}=\left[\cos\phi ,\sin\phi ,0\right] \end{array}$$

(11) $$\begin{array}{c} t_{x2}=t_{x1}+\pi \end{array}$$

(12) $$\begin{array}{c} R_{1}=\left[\begin{array}{ccc} \cos\beta & -\sin\beta & 0\\ \sin\beta & \cos\beta & 0\\ 0 & 0 & 1 \end{array}\right] \end{array}$$

(13) $$\begin{array}{c} R_{2}=\left[\begin{array}{ccc} 2\cos^{2}\phi -1 & 2\cos\phi \sin\phi & 0\\ 2\cos\phi \sin\phi & 2\sin^{2}\phi -1 & 0\\ 0 & 0 & -1 \end{array}\right]R_{1} \end{array}$$

**Figure 5:**
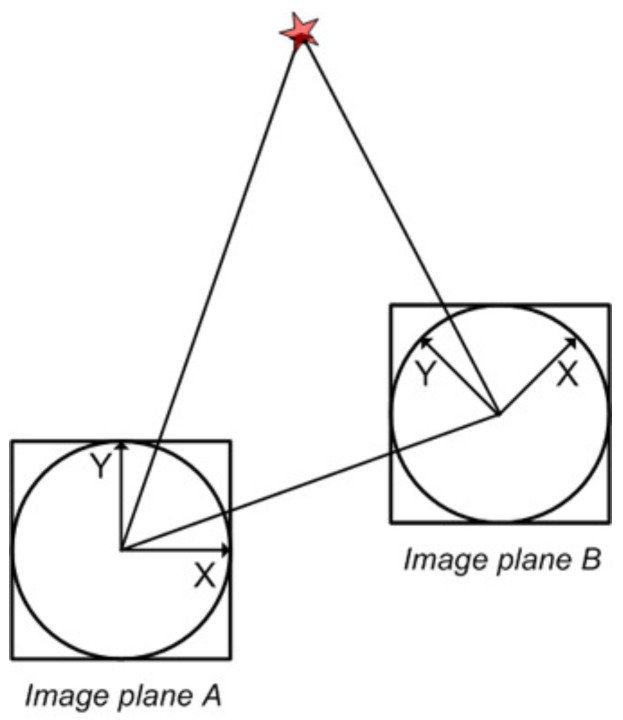
Interpretation of the valid solution pair ($R_{1},t_{x}$), on the plane XY.

Due to the projection nature of the omnidirectional sensor system, there is no longer an image plane pointing towards the 3D point direction. This leads to an interpretation procedure to discern between the four possible pairs: (($R_{1},t_{x1}$), ($R_{2},t_{x1}$), ($R_{1},t_{x2}$), ($R_{2},t_{x2}$)). The valid pair must return the backprojection of *X* in front of both cameras, that is, with both rays intersecting in the positive half of both camera reference systems, as shown in Figure 5, which represents the valid solution pair ($R_{1}$, $t_{x1}$) as per Equations (10) and (12).

### 2.3. Adaptive Matching

Having presented the motion recovery procedure, it is necessary to describe the design for the enhanced matching process. We seek to reinforce the final estimate and to avoid false positive inputs. This matching dynamically adapts to the non-linear noise and uncertainty characteristics of the system.

Again, relying on the epipolar constraint, defined in Equation (4), allows to delimit the search for matches on the expected epipolar ellipses for the omnidirectional camera system. In addition to this, current uncertainty errors are propagated to this process. The aim is to devise a procedure which accounts for dynamic changes on the uncertainty.

It is worth remembering that this omnidirectional visual odometry is intended to serve as the prior input for an EKF view-based SLAM approach. That is the main reason why we can take the most of the prediction stage of the EKF. In particular, it allows us to define a more realistic and dynamic threshold, $\delta (\hat{z_{t}})$, for the epipolar constraint, which now accepts deviations so as to prevent from false imparity when non-linearities are present, and also reduces the search for matches:

(14) $$x^{\prime T}\hat{E}x<\delta \left(\hat{z_{t}}\right)$$

Note that this new threshold depends on the EKF predicted motion, $\hat{z_{t}}=(\hat{\beta },\hat{\phi )}$, and it is also implicitly associated with the current uncertainty of the estimation of the current state vector of the system, $x_{v}\left(t\right)$, through the innovation $v_{t}$, and its covariance matrix $S_{t}$. Notice that the entire analytic structure of the EKF is divided into three stages:

- Prediction
  (15) $$\begin{array}{c} {\hat{x}}_{t+1|t}=f\left({\hat{x}}_{t|t},u_{t}\right) \end{array}$$
  (16) $$\begin{array}{c} {\hat{z}}_{t+1|t}=h\left({\hat{x}}_{t+1|t},x_{i}\right) \end{array}$$
  (17) $$\begin{array}{c} P_{t+1|t}=\frac{\partial f_{t|t}}{\partial x}P_{t|t}{\frac{\partial f_{t|t}}{\partial x}}^{T}+Q_{t} \end{array}$$
- Innovation
  (18) $$v_{t+1}=z_{t+1}-{\hat{z}}_{t+1|t}$$
  (19) $$S_{t+1}=\frac{\partial h_{t|t}}{\partial x}P_{t+1|t}{\frac{\partial h_{t|t}}{\partial x}}^{T}+R_{t+1}$$
- Update
  (20) $${\hat{x}}_{t+1|t+1}={\hat{x}}_{t+1|t}+K_{t+1}v_{t+1}$$
  (21) $$P_{t+1|t+1}=P_{t+1|t}-K_{t+1}S_{t+1}K_{t+1}^{T}$$
  (22) $$K_{t+1}=P_{t+1|t}H_{t}^{T}S_{t+1}^{-1}$$
  where the following terms are involved:
  - $f_{t}$: relation between the control input and the current state.
  - $u_{t}$: control input as the initial seed for the prediction.
  - $h_{t}$: relation between the observation and the current state.
  - $\frac{\partial f_{t|t}}{\partial x}$: jacobian of $f_{t}$ evaluated at the corresponding state.
  - $\frac{\partial h_{t|t}}{\partial x}$: jacobian of $h_{t}$ evaluated at the corresponding state.
  - $P_{t}$: covariance of the current uncertainty of the state.
  - $R_{t}$: covariance of the gaussian noise generated by the camera sensor.
  - $Q_{t}$: covariance of the gaussian noise generated by the internal odometers.
  - $K_{t}$: gain matrix of the filter which plays the role of weighting.

Eventually, $S_{t}$ represents an advantageous tool, from where to extract *σ* values for a predicted motion between poses, $\hat{z_{t}}$, with its final form:

(23) $$S_{t}=\left[\begin{array}{cc} \sigma_{\phi }^{2} & \sigma_{\phi \beta }\\ \sigma_{\beta \phi } & \sigma_{\beta }^{2} \end{array}\right]$$

**Figure 6:**
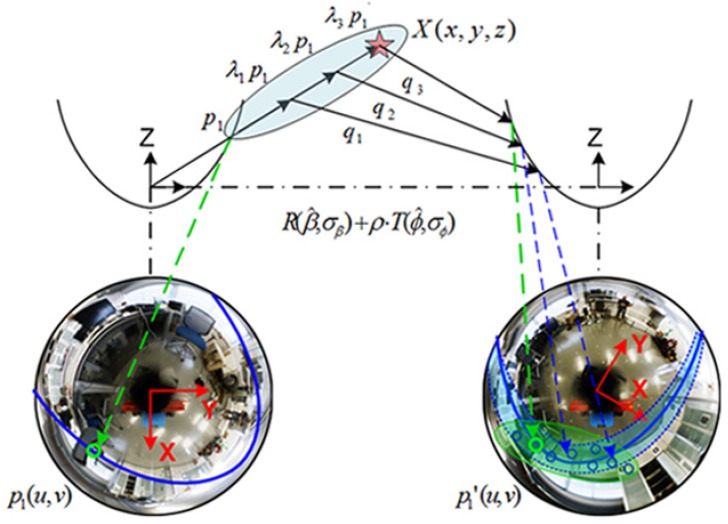
Adaptive matching: a feature point $p_{1}$ on the first image generates a multi-scaled distribution, $\lambda_{i}p_{1}$, to account for the lack of scale. Then it is transformed into $q_{i}$ on the second image through a rotation *R*∼N($\hat{\beta },\sigma_{\beta }$), a translation *T*∼N($\hat{\phi },\sigma_{\phi }$) and a scale factor *ρ*. Finally, $q_{i}$ is projected onto the second image plane to define a reduced area where matches must be searched. The circled points represent the projection of $\lambda_{i}p_{1}$ (in the first image), as $q_{i}$ (in the second image). The epipolar curve transforms into a reshaped area due to the effect of the uncertainty propagation and the motion prediction, as per $\delta (\hat{z_{t})}$ in Equation (14). Mahalanobis metric generates the final reshape on the feature descriptor space, denoted by the green area.

Notice that Figure 6 provides further detail about this process. The scale ambiguity is solved by means of a multi-scale distribution, and a predicted rotation and translation may be inferred as:

(24) $$\begin{array}{c} R∼N(\hat{\beta },\sigma_{\beta }) \end{array}$$

(25) $$\begin{array}{c} T∼N(\hat{\phi },\sigma_{\phi }) \end{array}$$

Therefore, candidate points must be found inside a restricted area, instead of a global search over the entire image. The current uncertainty reshapes and spreads the expected epipolar curve into an epipolar area, which implies more relaxed conditions when the uncertainty is high, and consequently false positives are more likely to appear. Ultimately, a Mahalanobis metric is applied on the visual descriptor space of the feature points, so as to reduce even more the search, as the denoted in the figure by the last green overlapping area. Note that this contribution allows us not to require a tracking process. Initially, the omnidirectional system provides a wider field of view. This fact makes increase the probability that dynamic objects are detected in the scene. Nonetheless, this adaptive matching proves to be a reliable tool so as to avoid dynamic objects as false positives.

## 3. View-Based SLAM

**Figure 7:**
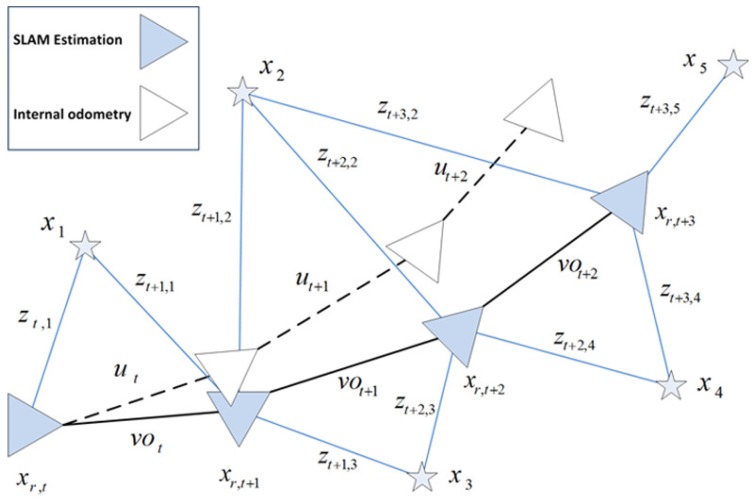
Graph-diagram of the view-based SLAM system. Colored items represent the estimation of the pose of the robot ($x_{r,t}$), at each *t*. Blank items represent the internal odometer estimation, $u_{t}$. A set of observed views in the map, $x_{n}$, are also indicated. The prior for the next SLAM state is defined by our omnidirectional visual odometry, $vo_{t}$. The observation measurement to the views are expressed by $z_{t,n}$.

In this section, we introduce our visual SLAM approach. In general terms, it can be synthesized as in Figure 7. More specifically, it consists of a dual 2D-3D map composed by a reduced set of omnidirectional views acquired at different poses, $x_{n}={\left(x,y,\theta \right)}_{n}^{T}$, along the path of the robot. Each *n* view compresses the visual information of an area of the environment by means of a set of *m* SURF feature points [38], $p_{n_{m}}$, with visual descriptors $d_{m}$, ∀m∈ [1,…,M]. The current pose of the robot at time *t* is expressed as $x_{r}={\left(x_{t},y_{t},\theta_{t}\right)}^{T}$. Therefore, the state vector comprises the current pose of the robot, $x_{r}$ and the set of views stored in the map, $x_{n}$, with the following 2D structure:

(26) $$x_{v}\left(t\right)={\left[\begin{array}{cccccc} x_{r} & x_{1} & \cdots & x_{n} & \cdots & x_{N} \end{array}\right]}^{T}$$

with each view n∈[1,…,N]. Then the state vector encodes a map constituted by a total number of *N* views.

**Figure 8:**
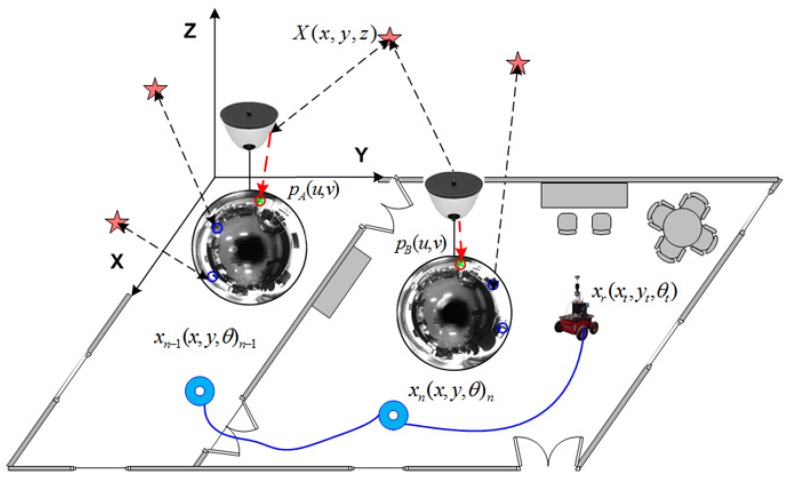
Dual 2D-3D map representation. Visual information is encoded on the 2D image plane by feature points in pixels (u,v), which are compressed on each view, $x_{n}$. These views are representative for specific areas of the environment with different visual appearance. The re-estimation of views implies a simpler re-estimation of larger number of 3D landmarks at once. The position where views were initiated in the map is indicated by circles. An example of real images is also included.

This arrangement benefits from the capability of omnidirectional images to encode large amounts of information due to their wide field of view. This consequently allows to reduce significantly the dimensions of the map, and so does the computational resources. Moreover the nature of this map allows to account for a dual 2D-3D representation. As it may be observed in Figure 8, the information is compressed on the 2D image frame by feature points. However, they express the same information that 3D landmark-based approaches [39,40]. Now it is not necessary to re-estimate the 3D pose of every landmark in the environment. Here, the single re-estimation of a view, as part of $x_{v}\left(t\right)$, already implies that process, being now much simpler. Note that the same 3D details can be reconstructed providing the retrieval of the scale factor, as explained in Section 2, with the initial prior of the odometer and then re-estimating by means of object recognition with known dimensions. Another positive outcome is that loop closure detection is not necessary under this context. The re-estimation of views, and the information they contain, produce an updated map estimation at each iteration step, so that loop closure and back-propagation is not required. Finally, it is worth noticing the role of each view $x_{n}$ in terms of information representation. They are representative of areas of the environment with different visual information, so that the robot can always localize itself anywhere, anytime.

Now, the view-based SLAM approach can be divided into three main stages, embedded within an EKF algorithm which uses the presented omnidirectional visual odometry as the prior input.

- (*i*) initialization of views in the map.
- (ii) observation model measurement.
- (iii) data association.

### 3.1. View Initialization

**Figure 9:**
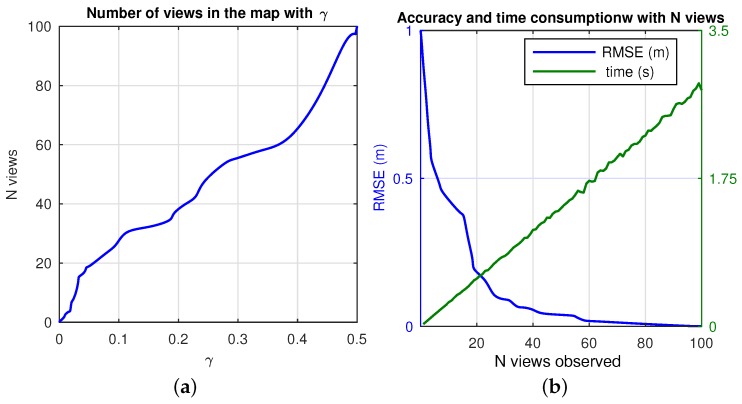
Effects of *γ* on the map estimation (mean values): (**a**) total number of views initiated in the map, *N* with *γ*; (**b**) accuracy and time consumption with the number of views observed in the map.

Here, the design of a balanced implementation to initiate new parts in the map is presented. These new parts should be understood as omnidirectional views. With the aim of establishing a reduced and compact map representation in terms of resources, we seek a strategy to produce scalable and feasible data for real applications. Hence the view initialization relies on a visual similarity ratio, *A*, which is experimentally defined as:

(27) $$A=k\frac{c}{p_{1}+p_{2}}<\gamma$$

being $p_{1}$ and $p_{2}$ the feature points detected on each image, and *c* the total matches, whereas *k* weights the current uncertainty at each *t*, so as to become adaptive to the particularities of each scenario. More specifically, a new view is initiated in the map whenever *A* stops meeting the experimental threshold, *γ*. That is, low values of *A* imply low visual similarity between a view in the map, $x_{n}$ and the view at the current pose, $x_{r}$, thus the need to initiate a new view in the map. This accounts for the encoding of relevant changes on the visual appearance of the scene, and thus a new view is initiated to that effect. This strategy seeks to produce an efficient arrangement of views which bounds the uncertainty and ensures convergence. For the experimental datasets used in this work [41], threshold *γ* ∈ [0.01–0.1]. This means that the expected visual appearance *A* ∼ [1–10]%. The effects on the final map are expressed in Figure 9. High values of *γ*, restrict the system to initiate more views in the map as seen in Figure 9a. The consequence is a more accurate estimation, however, this comes at a cost of computation time, as depicted in Figure 9b. Contrarily, when *γ* is set to a low value, the final map consists of a reduced set of views, with less accuracy, but also with less computation requirements. Hence the main effect lays on the tradeoff between accuracy on the estimation and time consumption. Notice that mean values are represented, where the accuracy is denoted as RMSE (m) in the final pose of the robot, and the time expresses the mean consumption at each iteration when *N* views are observed.

### 3.2. Observation Model

**Figure 10:**
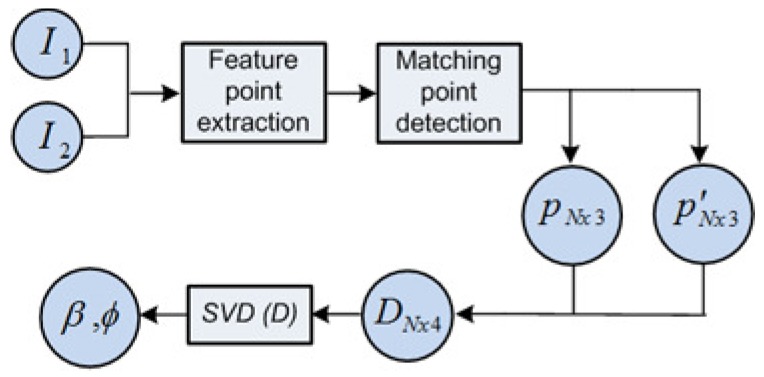
Observation model is embedded in the block diagram for the Scheme 1.

The basis of the observation model lies on the same idea sustaining the visual odometry approach, defined in Section 2. Similarly, the observation measurements are computed between the current robot’s image, at $x_{r}={\left(x_{t},y_{t},\theta_{t}\right)}^{T}$, and any *n* view within range in the map, $x_{n}$. Then, these measurements input the EKF to re-estimate the state, with the following structure:

(28) $$\begin{array}{c} z_{t,n}=\left(\begin{array}{c} \phi \\ \beta \end{array}\right)=\left(\begin{array}{c} \arctan\left(\frac{y_{n}-y_{t}}{x_{n}-x_{t}}\right)-\theta_{t}\\ \theta_{n}-\theta_{t} \end{array}\right) \end{array}$$

where ϕ and *β* are the relative angles expressing the bearing and orientation at which a view *n* is observed, as previously depicted in Figure 4. The workflow in this stage is synthesized in Figure 10, where:

- (*i*) feature points, *p* and $p^{\prime }$ are extracted from the omnidirectional images $I_{1}$ and $I_{2}$.
- (ii) the total *N* points input the SVD solver at once, as $D_{Nx4}$, namely in Equation (7).
- (iii) ultimately, they produce the single solution, as the observation measurement (*β*,ϕ).

### 3.3. Data Association

**Figure 11:**
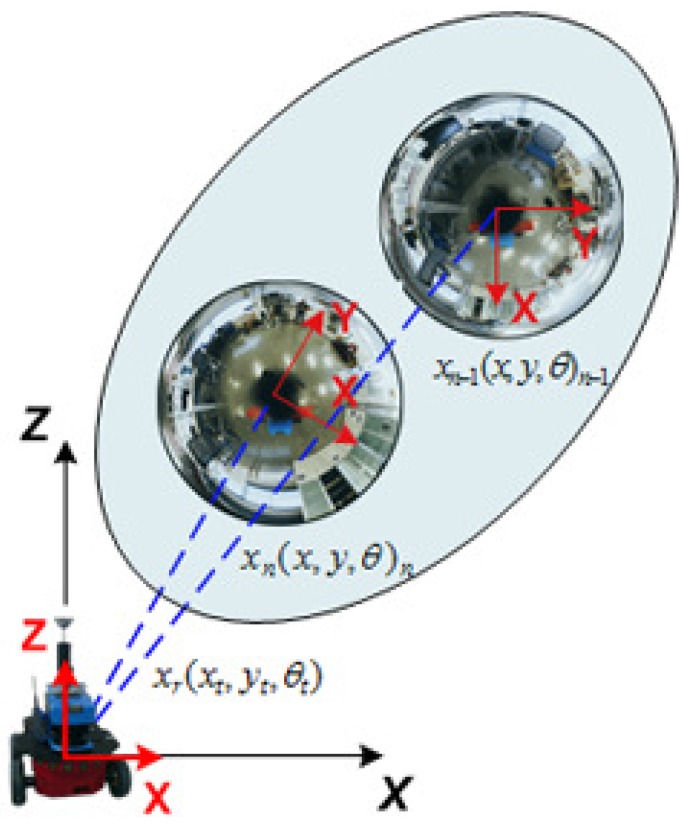
Data association problem with low parallax.

The data association problem usually reveals an issue in the presence of non-linearities [42,43], as observed in Figure 11. Here we address this problem via the evaluation of *A*, in Equation (27), among a set of candidate views in order to discern which of the observations in the set $z_{t}=\left[z_{t_{1}},...,z_{t_{B}}\right]$ at *t*, correspond to the correct view in the map. This set is extracted by using the maximum range at which the robot is able to compute observation measurement to a view. That is, views within the euclidean distance $D_{n}=\left|\right|{\left(x_{r}-x_{n}\right)}^{T}\left(x_{r}-x_{n}\right)\left|\right|$, where the notation corresponds to Equation (26). Next, the views with highest *A* are eventually chosen as the valid data association since they reveal the highest similarity with the current robot’s image. Then the observation measurement can be computed. At anytime *A* fails to meet with the threshold, a new view is initiated in the map. Algorithm 1 synthesizes this data association stage.

**Algorithm 1** Data Association**Require:** Inputs $x_{n_{i}}$ ∈ $x_{v}\left(t\right)$ ∀ *n*, where $x_{v}\left(t\right)$=[$x_{r}$, $x_{n_{1}}$, $x_{n_{2}}$, *…*
$x_{n_{N}}$] Can: Set of candidate views within range. Dassoc: Views maximizing similarity ratio *A*. $d_{max}$: Maximum range. $p_{1}$: feature points on robot’s image at $x_{r}$. $p_{2}$: feature points on view $x_{n}$. **for** i=1:N **do**  $D_{n_{i}}=\left|\right|{\left(x_{r}-x_{n_{i}}\right)}^{T}\left(x_{r}-x_{n_{i}}\right)\left|\right|$  **if**
$D_{n_{i}}$<$d_{max}$
**then**   New candidate to the subset:   Can=[$x_{n_{3}}$, $x_{n_{6}}$, …, $x_{n_{j}}$]  **end**
**if** **end**
**for** **for** j=1:length(Can) **do**  Extracting $p_{2}$ on $x_{n_{j}}$ ∈ Can  **if**
$A_{j}$=*k*
$\frac{c}{p_{1}+p_{2}}=max$
**then**   Dassoc=[$x_{n_{j}}$]  **end**
**if** **end**
**for** **return**
Dassoc

## 4. Results

In this section we concentrate on the real data experiments aiming at testing the improvements on the use of the presented omnidirectional visual odometry. We intend to use it as a prior input which substitutes the noisy data provided by the internal odometer of the robot. In addition to this, we modify the basis of our initial design in order to improve its performance. Furthermore, we present relevant outcomes on the robustness of a final SLAM application in mobile robotics, such as the view-based approach presented in Section 3.

The equipment used for the acquisition of data has already been presented by Figure 1a. It consists of a Pioneer P3-AT robot which is mounted with an omnidirectional camera, internal odometer and laser range finder, which produces a general ground truth [44,45] for comparison tasks. Table 1 synthesis the main characteristics of all the real scenarios where experiments were conducted. They correspond with indoor office and laboratory-like spaces. References to specific result figures and synthetic top view mockups for the layout have been also included.

### 4.1. Omndirectional Odometry

Firstly we present results solely based on the proposed omnidirectional visual odometry so as to assess its validity and suitability.

#### Dataset 1

**Figure 12:**
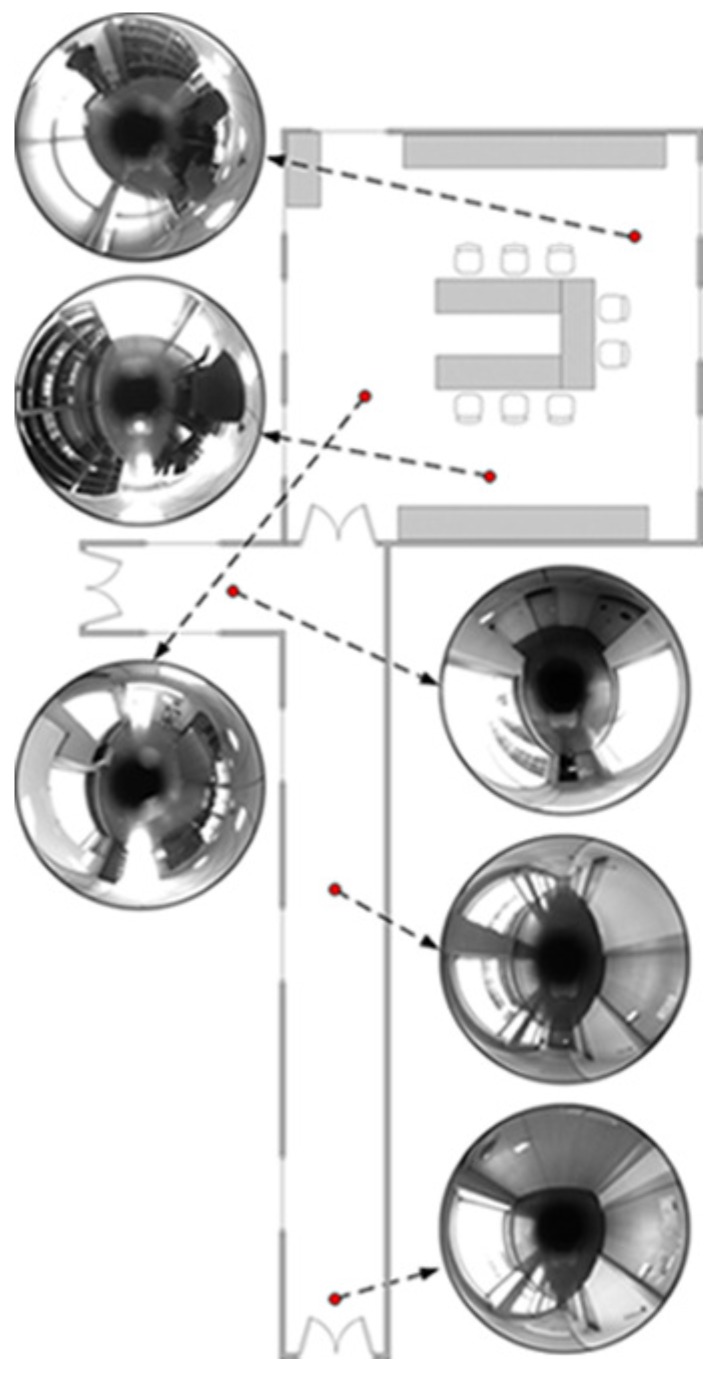
Mockup for Dataset 1. Six views of the environment are indicated.

**Figure 13:**
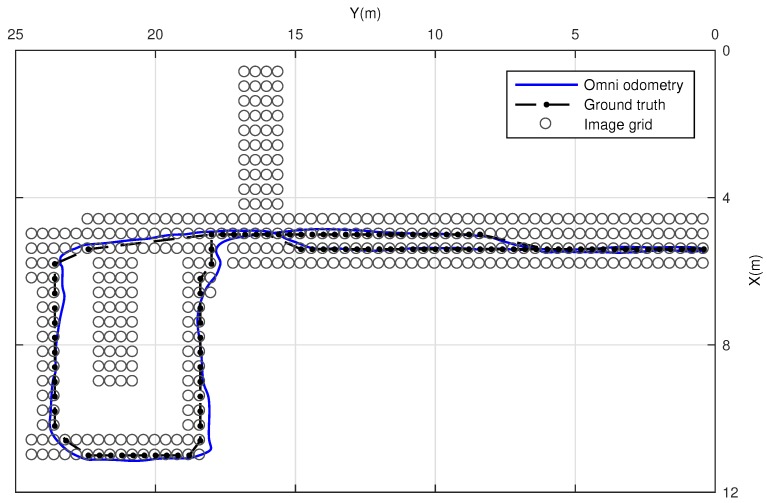
Omnidirectional odometry in Dataset 1. The estimated odometry is drawn in continuous line and the ground truth in dash-dotted line. Circles represent the entire grid of omnidirectional images.

**Figure 14:**
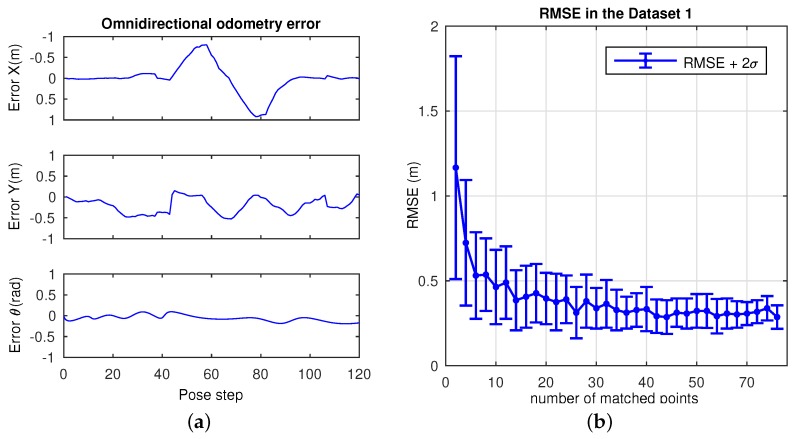
Omnidirectional odometry results in Dataset 1. (**a**) error in *X*, *Y* and *θ*; (**b**) RMSE (m) with 2*σ* versus the number of matched points.

This dataset is composed by a corridor with damaging and very changing lighting conditions due to large windows. A library room with meeting furniture, as modeled in Figure 12, also forms part of this layout. The data of this experiment was manually acquired over a grid in 381 positions, with a 40 cm of step size. Due to that fact, no internal odometer’s data is available to be presented. The main intention is to validate the suitability of this approach at first instance. In order to ensure robustness in terms of error, the experiment has been repeated 100 times, thus the results express mean values. Figure 13 presents the visual odometry results. The ground truth is drawn in dash-dotted line and the omnidirectional visual odometry estimation in continuos line. It can be noted that the topologic shape of the estimation demonstrates high resemblance with the ground truth. Figure 14 presents the obtained errors. Figure 14a compares them in *X*, *Y* and *θ*. Figure 14b plots the mean RMSE (m) at the last pose of the robot over the 100 repetitions versus the number of matched points considered for the motion recovery computation. Here, the evolution of the RMSE proves that the more number of matched points, the more accurate results.

### 4.2. Performance: Accuracy

Having presented preliminary results of visual odometry, the next stage to take into account is a further study on the precision and resource consumption of these measurements associated with the motion recovery presented in Section 2.2. Therefore we analyze the accuracy on the values (ϕ, *β*) and the time consumption under different circumstances and implications, such those related to the number of matched points. To that aim, we present different variants to the former SVD solver embedded in the motion recovery scheme, so as to improve the performance against non-linearities affecting the system.

#### 4.2.1. Solver 1

This is the kernel for the main approach of omnidirectional odometry, defined throughout this manuscript. In particular, its basis lies on the observation model already presented in Section 3.2, which is also represented by the diagram in Figure 10.

Figure 16a presents the accuracy obtained with Solver 1. The mean error on the angular solution is plotted versus the total number of matches, with frequency bars that represent a % of repetition of a specific number of matches found, out of the total within this experiment. Note that the scale on the *y*-axis expresses simultaneously angular error (degrees) and % of repetition of matching points. The *x*-axis indicates the number of matching points between images, determined by bins. Thus the resulting histogram is computed as:

$${\%}_{i}=\frac{frequency_{bin_{i}}}{\sum_{i=1}^{n}frequency_{bin_{i}}}$$

Figure 16a presents the evolution on the error in *β* and ϕ versus the number of matched points and their frequency of repetition (%). The precision on the estimated angles confirms the expected behavior: the more number of matched points, the better accuracy. Nonetheless, it is confirmed that estimations obtained with low number of matches, (i.e., 15) provide reliable results to work with real time applications.

#### 4.2.2. Solver 2

**Figure 15:**
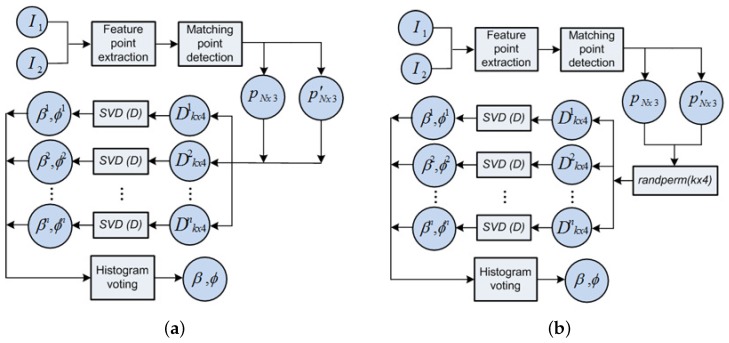
Block diagrams for the proposed solver variants: (**a**) Solver 2; (**b**) Solver 3.

This second variant divides the total number of matches between images into *n*-subsets which are next input into the SVD solver. As depicted in Figure 15a, n=N/k, with *k* the selected size of the subsets. In consequence, the solution consists of *n*-pairs of values (*β*, ϕ), denoted as ($\beta^{n}$, $\phi^{n}$). Finally an histogram voting with mean values is used to compute the final solution. The main purpose of this redesign is to spread possible false positives associated with the non-linear noise.

**Figure 16:**
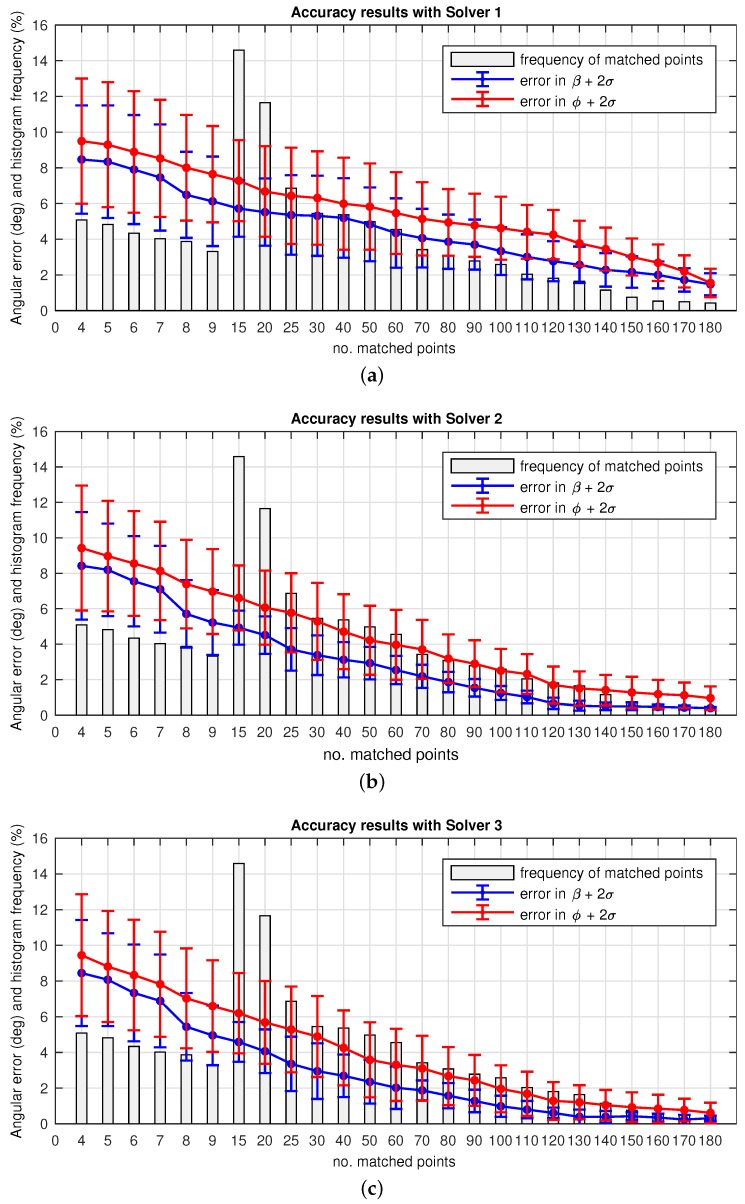
Omnidirectional odometry accuracy as the error in (*β*, *β*, ϕ) versus the number of matched points. (**a**) Solver 1; (**b**) Solver 2; (**c**) Solver 3. The bins represent subdivisions for the number of matched points detected. The frequency of repetition is presented as % out of the total.

Figure 16b presents the accuracy results for Solver 2. It is worth noticing that these results provide a more accurate estimation than in the previous solver. In particular, estimations obtained with only 9 matches are sufficient for its use in a real application. This confirms the mitigation of false positive, which now only bias the solution for a limited number of subsets. However, it is evident that these results come at the cost of computation time.

#### 4.2.3. Solver 3

Finally, this last variant randomly permutes the *n*-subsets presented in the previous Solver 2. This strategy seeks to enhance even more the robustness against the existence of false positive. To that aim, a combinatorial permutation is included in order to randomize and obtain a larger number of possible combinations for the *n*-subset of matched points. Figure 15b presents the diagram for this solver, where the introduction of this randomizer aids in the construction of each $D_{k\times 4}^{n}$.

Figure 16c presents the corresponding accuracy results. In this case it may be noted that the results provide the most accurate estimation. Nonetheless, they are quite similar to those provided by Solver 2. Besides this, the time consumption may become totally inviable for a normal use in a real-time application, as it becomes exponential. Higher number of matched points implies a computation effort for the generation of the permutation, which is definitely crucial when pursuing an appropriate balance between accuracy and time consumption. The next subsection evaluates this consideration about time.

### 4.3. Performance: Time Consumption

**Figure 17:**
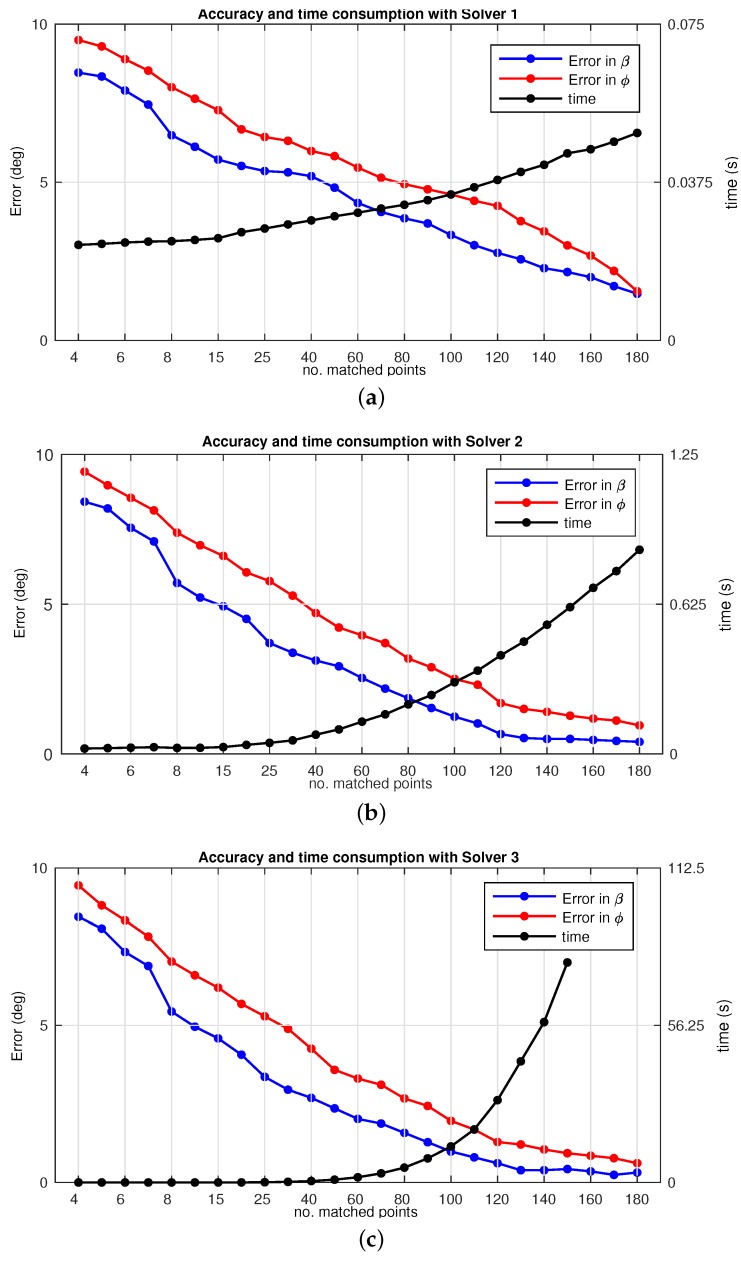
Time consumption and error in (*β*,ϕ) versus the number of matched points: (**a**) Solver 1; (**b**) Solver 2; (**c**) Solver 3.

According to the results presented above, an evaluation of computational costs is also required so as to complete a proper analysis on the three solvers. Hence Figure 17 presents a comparison for the time consumption. The error in the estimation is also overlapped in order to aid in the comparison. Figure 17a–c represent the same results for each specific solver, that is, Solver 1, Solver 2 and Solver 3 respectively. Note that these figures have been plotted separately due to the high difference on the right side axis scale.

Inspecting these figures reveals that large number of matched points is not highly necessary in order to retrieve a valid an accurate solution. It is obvious that the more number of matched points, the more accuracy on the estimation, however, Solver 2 and Solver 3 may only be suitable for certain applications, due to their associated time costs, as observed by the scale of the the right side axis.

### 4.4. SLAM Results

Once validated the suitability of the omnidirectional visual odometry to produce reliable and efficient results, we can move forward in order to test the behaviour of a SLAM application when the internal odometry is substituted by the proposed omnidirectional approach as the main prior input to the system. Notice that Solver 1 has been selected for the following experiments. According to the results presented above on performance, Solver 1 is the most appropriate for the dimensions of the datasets acquired, in order to get a proper tradeoff in terms of accuracy and time consumption.

#### 4.4.1. Dataset 2

**Figure 18:**
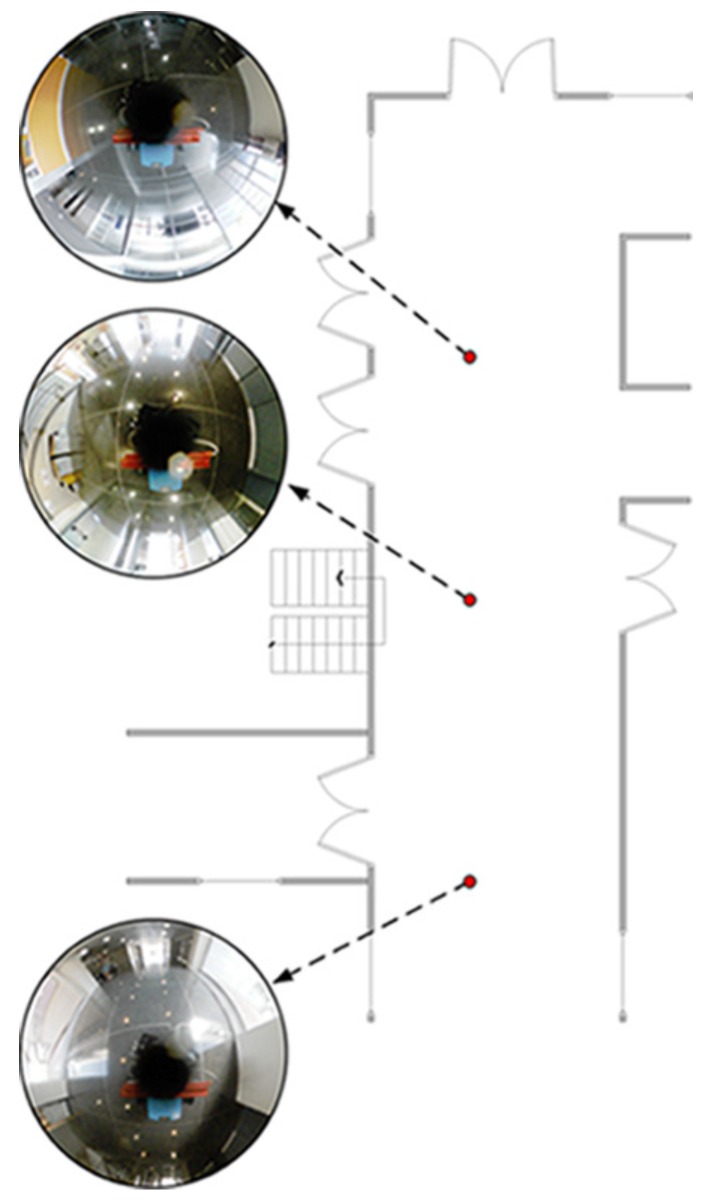
Mockup for Dataset 2. Three views of the environment are indicated.

**Figure 19:**
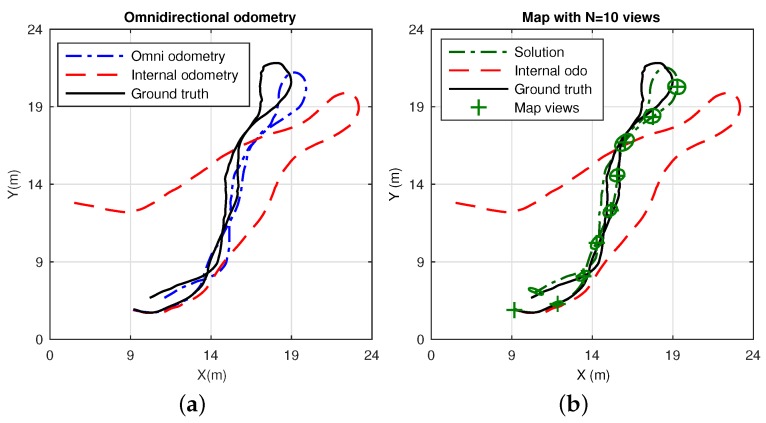
SLAM results in Dataset 2: (**a**) estimated omnidirectional visual odometry, drawn in dash-dotted line, ground truth in continuous line and internal odometry in dashed line; (**b**) estimated SLAM solution, in dash-dotted line, when the prior input is the omnidirectional visual odometry shown in (**a**). Again, the ground truth and internal odometry are also presented. The final map is constituted by *N* = 10 views, with their associated uncertainty ellipses.

**Figure 20:**
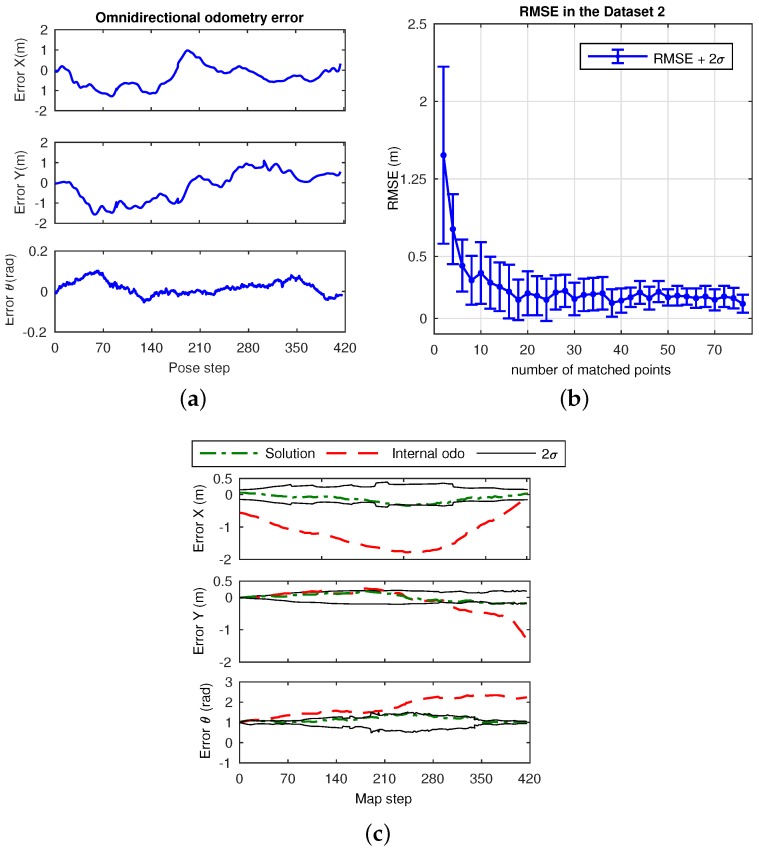
Error results in Dataset 2: (**a**) error in the omnidirectional visual odometry (Figure 19a), in *X*, *Y* and *θ*; (**b**) RMSE (m) for the omnidirectional odometry with 2*σ* versus the number of matched points; (**c**) error terms for the SLAM estimation presented in Figure 19b.

This dataset consists of a laboratory scenario with dynamic conditions, occlusions and obstructions. Its layout is represented by the mockup shown in Figure 18. The results are divided into estimation, in Figure 19, and error in Figure 20. Specifically, Figure 19a presents the omnidirectional odometry results, whereas Figure 19b shows the SLAM results obtained by means of the use of such odometry as the only prior. The appropriateness of this approach and its effectiveness to provide robust results is confirmed by inspection of the last figures, but it is also reinforced by Figure 20a–c, which respectively present the evolution of the error in the omnidirectional odometry, the RMSE versus the number of matched points, and the evolution of the error in the SLAM estimation.

#### 4.4.2. Dataset 3

**Figure 21:**
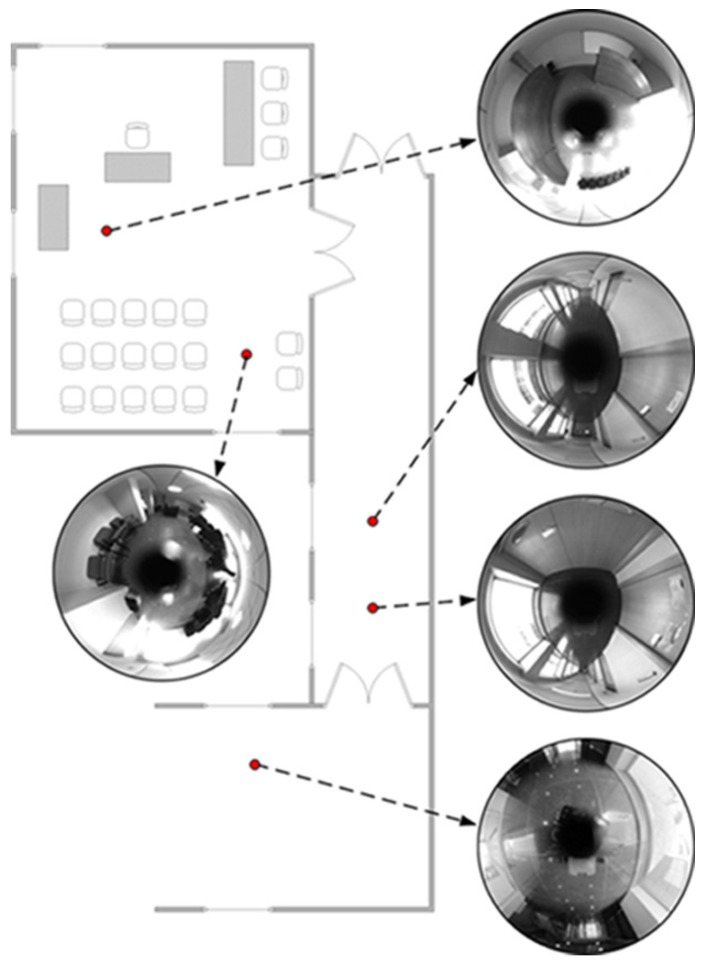
Mockup for Dataset 3. Five views of the environment are indicated.

**Figure 22:**
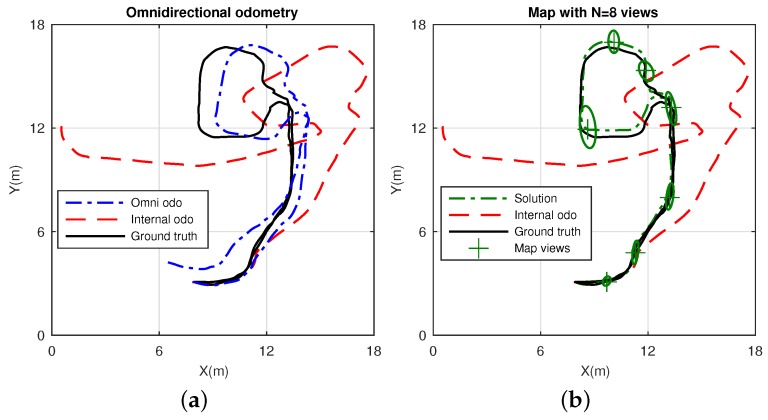
SLAM results in Dataset 3. (**a**) estimated omnidirectional visual odometry, drawn in dash-dotted line, ground truth in continuous line and internal odometry in dashed line; (**b**) estimated SLAM solution, in dash-dotted line, when the prior input is the omnidirectional visual odometry shown in (**a**). Again, the ground truth and internal odometry are also presented. The final map is constituted by *N* = 8 views, with their associated uncertainty ellipses.

**Figure 23:**
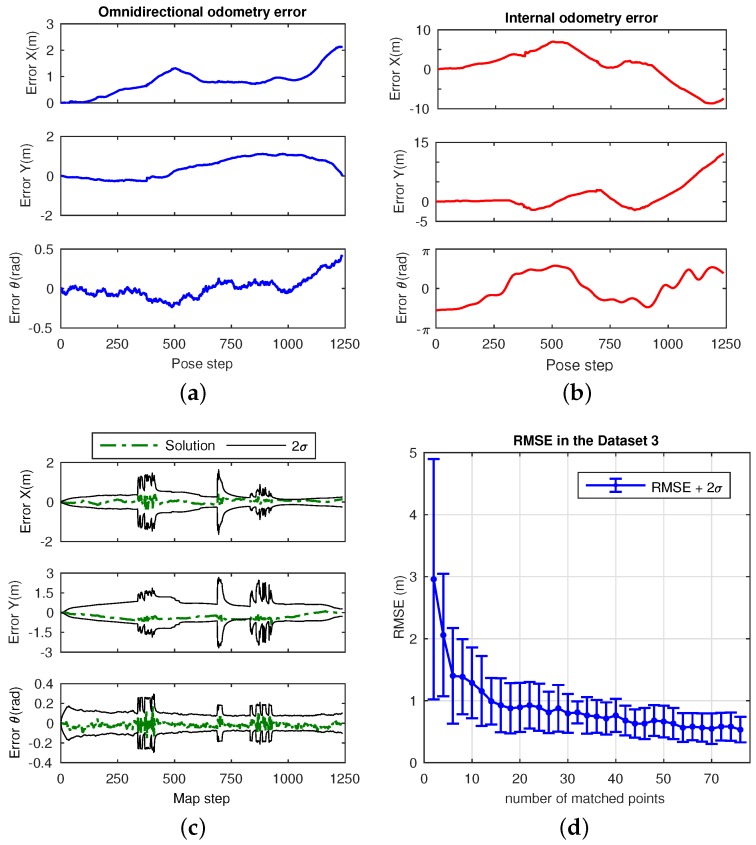
Error results in Dataset 3: (**a**) error in the omnidirectional visual odometry (Figure 22a) in *X*, *Y* and *θ*; (**b**) error in the internal odometry for comparison purposes; (**c**) error terms for the SLAM estimation presented in Figure 22b; (**d**) RMSE (m) for the omnidirectional odometry with 2*σ* versus the number of matched points.

This dataset corresponds to an office-like scenario with a corridor and large windows. Again, this causes undesired effects which affect the lighting conditions on the image acquisition. Finally, an office room is also considered in this scenario, as observed in its layout, in Figure 21. It is worth highlighting the larger dimensions of this scenario, since the objetive now is to validate the approach to work in a wider environment, and to extend the outcomes recently presented in the previous dataset. Figure 22 shows the results in terms of the obtained estimation, and Figure 23 shows the associated error. In particular, Figure 22a presents the omnidirectional odometry results, whereas Figure 22b shows the SLAM results obtained with the input of this omnidirectional odometry. Again, the suitability and precision are demonstrated, but also confirmed by Figure 23a,d,c, which respectively represent the error terms in the omnidirectional odometry, its associated RMSE versus the number of matched points, and the same variation on the error in the SLAM estimation. Note that Figure 23b has been represented separately for comparison purpose with the internal odometry, as the scale is considerably higher, being thus a fact that demonstrates a higher error.

## 5. Discussion

Once the results have been presented, in this section we provide a further discussion on the main aspects extracted from these results.

Firstly, as a preliminary outline from the experiments shown in Figure 14, this omnidirectional visual odometry approach demonstrates that the relative angles *β* and ϕ obtained from the motion recovery, are valid and suitable for final applications, as per the reliable solution compared to the reference ground truth.

The next experimental sets pointed out several aspects in terms of performance. Figure 16 comprises several facts to highlight as a result of the proposed variants to overcome false positives:

- (*i*) Better results are obtained at any solver case with higher number of matched points considered in order to compute the motion recovery. This implies a considerable increase on the computation time, which may become inviable.
- (ii) Particularly, Solver 2 and Solver 3 are liable to require such time efforts, as observed in Figure 17. Despite this fact they provide useful outcomes in order to mitigate false positives.
- (iii) Overall, a well devised tradeoff solution may be reached, depending on the final application. Solver 1 may provide sufficient accuracy at a low time consumption, for time demanding applications. The other two solver proposals can be advantageous under cases where the real need is to avoid false imparity, regardless the time consumed.

Regarding the final experiments within the context of a SLAM system, Figure 19 and Figure 22 confirm the suitability of the approach to work with real data environments, where the fusion of the proposed omnidirectional visual odometry into the system reveals appropriate and enhanced results in terms of error, in contrast to the internal odometry, as pointed out in the analyzed results in Figure 20 and Figure 23.

## 6. Conclusions

This article has presented a robust approach to omnidirectional visual odometry which is worth operating as a feed-forward prior input for a view-based SLAM model within a mobile robotic application. The main purpose was to strengthen its capability to deal with those harmful effects associated with non-linearities introduced by non-systematic terms in the internal odometer, which very often become a hazardous issue to ensure convergence in the estimation. The final outcome is a robust omnidirectional visual odometry which can substitute the internal odometry.

The implementation has considered the adaption of the epipolar constraint to the omnidirectional geometry of the sensor, together with an adaptive matching with uncertainty considerations, so as to reduce those non-linear effects in terms of false positives by establishing a limited pixel are for the feature search. A set of real experiments have been conducted in order to test the appropriateness of this approach and its performance. Moreover, three variants of the former model have been designed in order to evaluate alternatives to improve its behaviour. The results reveal the strengths of each one according to the requirements of the final application. Thus allowing to select the more suitable as an efficient and balanced tradeoff oriented to the particularities of the environment and the application specifications. Finally, this omnidirectional odometry has been assessed as the only prior input for a SLAM system. The results demonstrate its suitability and reliability to produce a robust estimation.

To conclude, this approach proves its feasibility to become a trustworthy input for visual-based SLAM systems, being capable to generate real-time oriented results.

## Figures and Tables

**Table 1:** Dataset characteristics.

Dataset Characteristics				
Dataset	No. Images	Distance	Figures	Mockup
Dataset 1	121	48.4 m	Figure 13 and Figure 14	Figure 12
Dataset 2	416	41.6 m	Figure 19 and Figure 20	Figure 18
Dataset 3	1238	123.8 m	Figure 22 and Figure 23	Figure 21

## Abbreviations

The following abbreviations are used in this manuscript:

SLAM	Simultaneous Localization and Mapping
EKF	Extended Kalman Filter
SVD	Single Value Decomposition
RMSE	Root Mean Squar Error
